# Prevalence and Predictors of Potentially Inappropriate Prescribing in Older People Receiving Home Health Care in Saudi Arabia According to the American Geriatrics Society Beers Criteria 2019

**DOI:** 10.3390/healthcare12202028

**Published:** 2024-10-12

**Authors:** Wael Y. Khawagi, Abdullah A. Alshehri, Ziyad M. Alghuraybi, Abdullah K. Alashaq, Rayan A. Alziyadi, Ahmed I. Fathelrahman

**Affiliations:** 1 Department of Clinical Pharmacy, College of Pharmacy, Taif University, Taif 21944, Saudi Arabia; a.aalshehri@tu.edu.sa (A.A.A.); aihassan@tu.edu.sa (A.I.F.); 2 Pharmaceutical Care Department, Ministry of National Guard Health Affairs, Jeddah 11426, Saudi Arabia; ziyad.m.basha@gmail.com; 3 College of Pharmacy, Taif University, Taif 21944, Saudi Arabia; abdullah.alashaq.31@gmail.com (A.K.A.); rayanotb1419@gmail.com (R.A.A.)

**Keywords:** inappropriate prescribing, prescribing safety, home care, beers criteria, older patients, Saudi Arabia

## Abstract

Background/Objectives: Potentially inappropriate prescribing (PIP) is a common health problem in older adults and is associated with negative health outcomes such as the occurrence of adverse drug events. Several studies have been conducted in different countries and settings to assess the prevalence of PIP, including in Home Care Services. However, data on the prevalence of PIP in home-care services in Saudi Arabia are limited. This study aimed to evaluate PIP use among older patients receiving home healthcare services in Saudi Arabia and to identify the predictors and commonly implicated medications.; Methods: A cross-sectional study was conducted over an 8-month period between January and August 2023. Data were collected from the medical records of patients older than 65 years who were currently receiving home health care services at King Faisal Hospital in Taif City, Saudi Arabia. PIPs were identified using the 2019 updated Beers Criteria.; Results: A total of 375 patients were included. Out of these, 285 PIPs were identified, of which 219 patients (58.4%) received at least one PIP. The most common therapeutic class associated with the PIPs was gastrointestinal medications (66.3%). Patient age and number of medications were significant predictors of PIP.; Conclusions: Our study found a high prevalence of PIP among elderly patients receiving home health care in Taif, Saudi Arabia. This study highlights the need for improved patient data automation and implementation of the Beers criteria to prevent PIPs in the future.

## 1. Introduction

As the population ages, the use of home health care services has become increasingly common, especially among older adults [1]. Home health care offers a convenient and cost-effective way for older people to receive care in the comfort of their own homes, while also reducing the burden on hospitals and other health care facilities [1,2,3,4]. However, older people receiving home health care may be at risk of potentially inappropriate prescribing (PIP), which can result in adverse drug reactions, hospitalizations, and negative health outcomes [4,5].

PIP in older people has become a global healthcare concern because of its link to negative health outcomes such as adverse drug events (ADEs) [6,7,8,9]. Medicines are considered appropriate for general adults when they have a clear, scientific evidence-based indication, are well tolerated by most patients, and are cost effective. Patients aged > 65 years with complex and multiple morbidities are usually excluded from randomized clinical trials; thus, prescribing decisions in the older population are frequently made in the absence of scientific evidence [6]. Older people are more likely to suffer from chronic diseases and disabilities, negatively impacting their quality of life. According to the Saudi General Authority for Statistics, in 2022, more than 1.19 million people in Saudi Arabia were older than 65 years [10].

Previous studies assessing the prevalence of PIP in home care settings have been conducted in different countries and settings [5,11,12]. A study in Qatar assessed the prevalence of PIP in home care settings. The study reported that 65% of the patients received at least one PIP, 25% received two PIPs, 8% received three PIPs, and 2% received four PIPs [5]. In the United States, a study assessed the prevalence of PIP in the home care setting. The study included 3124 patients: 38% of them had at least one PIP and 26% had at least one PIP with potential adverse outcomes of high severity [11]. A study in Europe assessed the prevalence of PIPs in the home care setting, involving 2707 patients: 535 patients were found to have PIP, 95% of them received at least one medication and polypharmacy (≥6). The study was conducted in eight countries; the highest prevalence (41.1%) was documented in Czech Republic and the lowest prevalence (5.8%) was documented in Denmark. They found 10 common medications, of which 2 were used in all countries (diazepam and amitriptyline) [12]. Despite this extensive body of research, no studies to date have explored the prevalence or predictors of PIP among older adults receiving home health care in Saudi Arabia. Given the unique healthcare landscape and population demographics, it is essential to investigate this issue locally to better understand its scope and contributing factors. Saudi Arabia’s aging population presents a growing need for targeted healthcare strategies [10]. Therefore, this study aimed to investigate the prevalence and predictors of PIP in this population and identify the most commonly prescribed medications that may contribute to PIP.

## 2. Materials and Methods

### 2.1. Study Design, Setting, and Population

This study had a retrospective cross-sectional design and was conducted over an 8-month period from January to August 2023. Data were obtained from the electronic medical records of individuals receiving home care health services at King Faisal Hospital in Taif, Saudi Arabia. The study included patients aged 65 years or older who were actively prescribed at least one medication during the study period. Patients were excluded if they were no longer receiving home care services during the study period or were not prescribed any medications.

### 2.2. Data Collection

A standardized data collection form was used to systematically gather patient information from the medical records. These records were maintained in a paper format at King Faisal Hospital. The collected information included demographic details (such as age, sex, and nationality), comorbidities, and medications prescribed to each patient.

### 2.3. Outcomes

The primary outcome of interest was the identification of PIPs in older patients. To assess this, the study utilized the 2019 updated American Geriatrics Society (AGS) Beers Criteria, a widely recognized tool for identifying medications posing risks to older adults [13]. Medications were classified into therapeutic classes according to the British National Formulary (BNF), providing a standardized system for categorizing medications according to their pharmacological properties and indications [14].

### 2.4. Data Analysis

Following data collection, information was entered into Microsoft Excel by one researcher and data entry was independently verified by another researcher to ensure accuracy. Descriptive statistics, including the mean, standard deviation, and relative and absolute frequencies, were used to summarize the data. Univariate and multivariate binary logistic regression analyses were performed to identify risk factors associated with PIPs. The results were reported as crude odds ratios (ORs) and adjusted odds ratios (aORs), with their corresponding 95% confidence intervals (95% CI). A variable was considered an independent predictor when the aOR was computed via multivariate analysis and the finding was found to be statistically significant and the statistical significance was judged at *p* value ≤ 0.05. A descriptive analysis was performed using Microsoft Excel (Version 16.78) and a logistic regression analysis was conducted using Stata 16.

### 2.5. Ethical Considerations

Ethical approval for the study was granted by the research and ethical committees at Taif University (Approval Number: 44-108) and King Faisal Medical Complex (Approval Number: 2023-B-26). Data were handled with utmost care to safeguard patient confidentiality. There was no direct contact with the patients, and the data were extracted from the medical records only; thus, no patient consent was required.

## 3. Results

### 3.1. Patient Characteristics

In total, 375 patients were included in the study, most of whom were female (*n* = 252). The patients’ mean age (±SD) was 80 (±8.9) years, they received home health care service for 2.2 years (±1.8) and were prescribed 7.1 medications (±2.7) on average. The most common comorbidities among the patients were hypertension (81.3%) and diabetes mellitus (55.7%). Table 1 shows the demographic and clinical characteristics of the study population.

### 3.2. Prevalence of Potentially Inappropriate Prescribing

**Figure 1:**
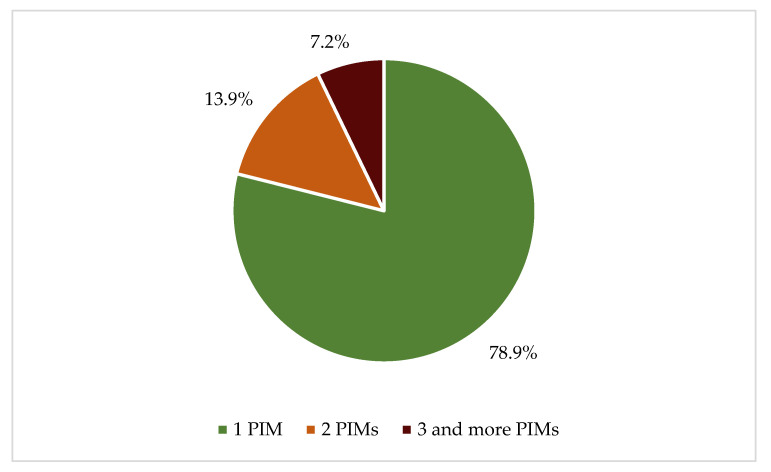
Distribution of patients who suffered PIPs by number of PIPs.

A total of 288 PIPs were identified, with 223 patients (59.5%) receiving at least one PIP. Among these patients, 176 (78.9%) received one PIP, 31 (13.9%) received two PIPs, and 16 (7.2%) received three PIPs or more (Figure 1).

### 3.3. Potentially Inappropriate Prescribing Characteristics

**Figure 2:**
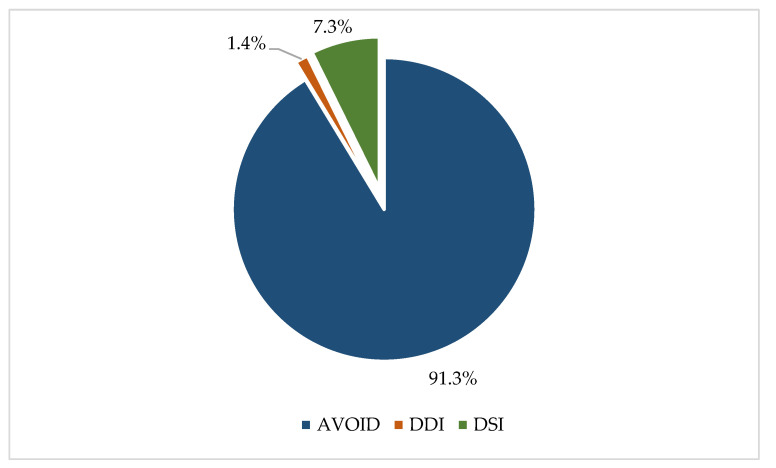
Distribution of patients who suffered PIPs by type of PIPs.

According to the Beers criteria, 91.3% (*n* = 263) of the identified PIP were classified as ‘Avoid in older adults’, with 7.3% (*n* = 21) classified as ‘drug-syndrome interaction (DSI)’, and 1.4% (*n* = 4) classified as ‘drug-drug interaction (DDI)’ (Figure 2).

**Figure 3:**
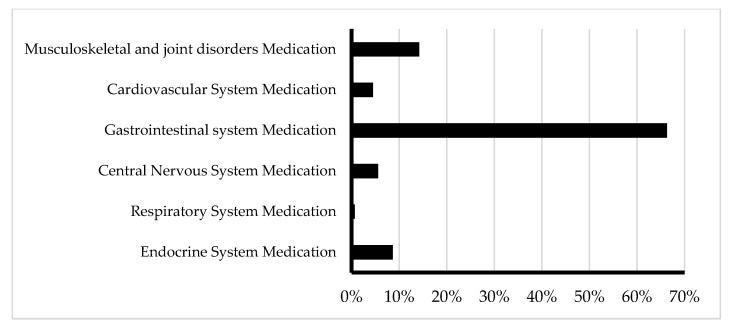
Medications commonly implicated in PIP by body system.

Medications commonly implicated in PIP by body system included gastrointestinal medications (66.3%), musculoskeletal and joint (14.2%), endocrine medications (8.7%), CNS drugs (5.6%), cardiovascular (4.5%), and respiratory (0.7%) (Figure 3). Table 2 shows common medications by therapeutic class.

### 3.4. Predictors of Potentially Inappropriate Prescribing

The prevalence of PIPs was lower in males (57.7%, adjusted odds ratio [aOR] 0.1; 95% CI 0.5–1.4) compared to females (60.3%). Among age groups, the highest PIP prevalence was observed in the 75–79 years group (67.9%, aOR 2.4; 95% CI 1.1–5.4), followed by the 70–74 years group (66.7%, aOR 2.4; 95% CI 1–5.7), 85–89 years group (60.8%, aOR 2.6; 95% CI 1.1–6.3), ≥90 years group (57.9%, aOR 2.1; 95% CI 0.9–4.9), 80–84 years group (54.9%, aOR 1.5; 95% CI 0.7–3.3), and 65–69 years group (43.2%). The prevalence of having 10 or more medications was highest (81.2%, aOR 21.8; 95% CI 6.2–77.0), followed by 7–9 medications (64.2%, aOR 8.4; 95% CI 2.7–25.5), 4–6 medications (52.4%, aOR 3.7; 95% CI 1.3–10.9), and 1–3 medications (18.5%). Similarly, the prevalence of having three comorbidities was highest (66.9%, aOR 1.9; 95% CI 0.7–4.8), followed by four or more comorbidities (60.3%, aOR 0.8; 95% CI 0.3–2.2), two comorbidities (55.2%, aOR 1.4; 95% CI 0.5–3.5), and one comorbidity (37.9%). Therefore, age groups and the number of medications were significant predictors of PIP in this sample (Table 3).

## 4. Discussion

This study aimed to evaluate PIP in older patients receiving home healthcare services in Saudi Arabia. The study was based on the 2019 updated AGS Beers criteria. The AGS Beers criteria were the first ever established criteria for assessment of inappropriate prescribing, and they have been well-recognized and widely used in the literature [15,16,17,18]. The findings revealed a substantially high prevalence at a rate of 53.2%. Gastrointestinal medications are commonly implicated in PIP. Age groups and number of medications were the only significant predictors of potentially inappropriate prescriptions in this study.

Previously published studies conducted in Qatar, the United States, and Europe reported lower prevalence, ranging from 20% to 40%. For instance, a study conducted in Qatar found a prevalence of 38.2% for at least one PIP according to the 2012 Beers criteria list [5,19]. Similarly, a study in the United States reported a prevalence of 38% for at least one PIP according to the 2003 Beers criteria list, with polypharmacy being identified as the main predictor [11,20]. A study from the United Arab Emirates involving older patients discharged from a hospital had assessed PIP using 2019 Beers criteria and reported a rate of 34.7% [21]. The lowest prevalence of PIP was reported in Europe at 20%, where polypharmacy, poor economic status, and anxiolytics were identified as the main predictors [12]. Rates of PIP and PIP differed in the literature by country, by patients’ characteristics such as age (e.g., older, middle aged, or children), by assessment tool, and by type of settings. For example, a study conducted in Malaysia using a combination of 2015 beers and 2015 STOPP criteria assessment revealed rate differences between inpatients and outpatients, where inpatients experienced higher rates [22]. In a study from Poland, the authors compared a couple of assessment tools, including the 2019 Beers criteria and STOPP/START version 2, which revealed variability in the detection capacity. Proton-pump inhibitors (PPIs) are the most common class of medication used for PIP. The STOPP, EU(7)-PIP, and Beers criteria revealed cases of inappropriate prolonged PPI use, whereas the Amsterdam tool identified cases in which PPIs should have been prescribed, but not [16].

The high prevalence of PIP in home healthcare settings can be attributed to multiple factors including the presence of various medical conditions and polypharmacy. Our study identified polypharmacy and age as the main predictors of PIP. In the regression analysis, patients aged 70–79 years were significantly more likely to experience PIP compared to those aged 65–69 years, with the highest risk observed in the 75–79 years age group. This suggests that PIP risk increases with age, particularly between 70 and 79 years, where physiological changes and multiple conditions make prescribing more challenging. Polypharmacy, in particular, has been consistently identified as a common cause of inappropriate prescribing in several studies [5,11,12]. Studies conducted among middle-aged patients confirmed the same finding regarding polypharmacy [23]. Our study found that patients taking 10 or more medications were 21.8 times more likely to have a PIP compared to those taking 1–3 medications. This strong association highlights the risk that polypharmacy poses in older patients, increasing the likelihood of inappropriate prescribing. In addition to these, it is important to acknowledge other age-related changes in pharmacokinetics—such as reduced metabolism, renal clearance, and altered drug distribution—can lead to drug accumulation. The decline in homeostatic mechanisms further reduces medication tolerance, increasing the risk of PIPs.

The alarming PIP rates in our study should initiate an ambitious plan that includes a combination of evidence-based strategies and interventions. Only approaches that have proven to be effective should be adopted. According to a systematic review by Kroon et al., multifaceted interventions were the most frequently effective strategies aiming at reducing inappropriate prescribing, followed by interventions containing additional diagnostic tests (antibiotics), computer interventions, audit and feedback, patient-mediated interventions and multidisciplinary (team) approach [24]. Surprisingly healthcare professionals’ education was the least frequently effective intervention. However, these reviews covered a wide range of drugs, populations, and settings. A study specifically evaluating the effectiveness of staff education on the appropriateness of prescribing in a cluster of nursing homes for older people in Serbia revealed a significant reduction in the rates of PIP using the STOPP, START, and Beers criteria [25].

As the first study of its kind in Saudi Arabia, our findings are expected to provide a valuable reference for future research on inappropriate prescribing practices. However, it is important to acknowledge that our study was conducted at a single medical center in Taif, which limits the generalizability of our results to other settings. A notable limitation was the manual documentation of patient files, which hindered efficient access to necessary information. The use of a retrospective data collection approach from medical records has known limitations such as the potential for collecting inaccurate or missing information. Future studies should address these limitations by involving multiple healthcare centers and implementing improved data management systems to enhance data completeness and accessibility.

To address the high prevalence of PIPs among older patients, healthcare settings must embrace the integration of the Beers criteria, which offers evidence-based guidelines for medication use in this vulnerable population. By adopting these criteria across all healthcare settings, clinicians can make informed decisions when selecting medications for older patients, thereby reducing the risk of adverse drug events. Furthermore, immediate collaboration with physicians and other healthcare professionals is crucial to implement interventions aimed at reducing PIP. Educational programs tailored to prescribers, as well as ongoing medication reviews, can significantly improve prescribing practices. In addition, quality improvement initiatives focusing on medication safety should be prioritized in home healthcare settings. These actions will complement further research into the specific causes of PIPs in Saudi Arabia and will be instrumental in crafting sustainable interventions to improve patient outcomes.

## 5. Conclusions

This study found a high prevalence of potentially inappropriate prescribing use among elderly patients receiving home healthcare services in Taif, Saudi Arabia. Polypharmacy and age were identified as significant contributors to inappropriate prescription. This study highlights the need for improved patient data automation and implementation of the Beers Criteria to prevent PIP in the future.

## Figures and Tables

**Table 1:** The demographic and clinical characteristics of the study sample.

Demographic and Clinical Characteristics	*n* (%)	Mean (SD), Range
Sex		
Female	252 (67.2)	—
Male	123 (32.8)	—
Age (years)	—	80.0 (8.9), 65–108
No. of prescribe medication	—	7.1 (2.7), 1–16
Home health care serves stay (years)	—	2.2 (1.8), 0–20
Comorbidities		
Hypertension	305 (81.3)	—
Diabetes mellitus	209 (55.7)	—
Ischemic Heart Disease.	80 (21.3)	—
Osteoarthritis.	77 (20.5)	—
Stroke	49 (13.1)	—
Benign Prostatic Hyperplasia	48 (12.8)	—
Dementia	36 (9.6)	—
Thyroid Disorders	34 (9.1)	—
Asthma	28 (7.5)	—
Gastric or duodenal ulcer	28 (7.5)	—
Heart Failure	24 (6.4)	—
Dyslipidaemia	20 (5.3)	—
Atrial fibrillation	18 (4.8)	—

**Table 2:** Therapeutic classes commonly associated with potentially inappropriate prescribing.

Potentially Inappropriate Medication Class	Number (%)
Proton-pump inhibitors	187 (64.9)
Digoxin	40 (13.9)
Sulfonylurea	14 (4.9)
Nonsteroidal anti-inflammatory drugs	10 (3.5)
Antipsychotics	8 (2.8)
Renin-angiotensin system (RAS) inhibitors	5 (1.7)
Barbiturates	4 (1.4)
Nondihydropyridine calcium channel blockers	5 (1.7)
Selective serotonin reuptake inhibitor	3 (1)
Insulin	2 (0.7)
Antispasmodics	2 (0.7)
Growth hormone	1 (0.3)
Prokinetic agents	1 (0.3)
Antiparkinsonian	1 (0.3)
Nonbenzodiazepine	1 (0.3)

**Table 3:** Prevalence and determinants of potentially inappropriate prescribing.

Variable	PIP Prevalence	OR (95% CI)	aOR (95% CI)
Sex			
Female	60.3%	Reference	
Male	57.7%	0.9 (0.6–1.4)	0.1 (0.5–1.4)
Age group *			
65–69	43.2%	Reference	
70–74	66.7%	2.6 (1.2–5.9)	2.4 (1–5.7)
75–79	67.9%	2.8 (1.3–5.9)	2.4 (1.1–5.4)
80–84	54.9%	1.6 (0.8–3.4)	1.5 (0.7–3.3)
85–89	60.8%	2 (0.9–4.6)	2.6 (1.1–6.3)
≥90	57.9%	1.8 (0.8–4)	2.1 (0.9–4.9)
Number of medications *			
1–3	18.5%	Reference	
4–6	52.4%	4.9 (1.7–13.5)	3.7 (1.3–10.9)
7–9	64.2%	7.9 (2.8–22.2)	8.4 (2.7–25.5)
≥10	81.2%	19 (6–59.5)	21.8 (6.2–77.0)
Number of comorbidities			
1	37.9%	Reference	
2	55.2%	2 (0.9–4.7)	1.4 (0.5–3.5)
3	66.9%	3.3 (1.4–7.7)	1.9 (0.7–4.8)
≥4	60.3%	2.5 (1.1–5.7)	0.8 (0.3–2.2)

Regression analyses were conducted assuming at least one PIP, * Significant independent predictors, OR: crude odds ratio, aOR: adjusted odds ratio.

## Data Availability

The datasets used and analyzed during the current study are available from the corresponding author on reasonable request.

## References

[1] Almoajel A., Al-Salem A., Al-Ghunaim L., Al-Amri S. (2016). The quality of home healthcare service in Riyadh Saudi Arabia. Asian J. Nat. Appl. Sci..

[2] Rusli K.D.B., Tan A.J.Q., Ong S.F., Speed S., Lau Y., Liaw S.Y. (2023). Home-based nursing care competencies: A scoping review. J. Clin. Nurs..

[3] Zolot J. (2018). At-home hospital care reduces readmissions and length of stay, enhances patient satisfaction. AJN Am. J. Nurs..

[4] Al-Surimi K., Al-Harbi I., El-Metwally A., Badri M. (2019). Quality of life among home healthcare patients in Saudi Arabia: Household-based survey. Health Qual. Life Outcomes.

[5] Alhmoud E., Khalifa S., Bahi A.A. (2015). Prevalence and predictors of potentially inappropriate medications among home care elderly patients in Qatar. Int. J. Clin. Pharm..

[6] O’connor M.N., Gallagher P., O’mahony D. (2012). Inappropriate prescribing. Drugs Aging.

[7] Bradley M.C., Motterlini N., Padmanabhan S., Cahir C., Williams T., Fahey T., Hughes C.M. (2014). Potentially inappropriate prescribing among older people in the United Kingdom. BMC Geriatr..

[8] Rancourt C., Moisan J., Baillargeon L., Verreault R., Laurin D., Gregoire J.P. (2004). Potentially inappropriate prescriptions for older patients in long-term care. BMC Geriatr..

[9] Mekonnen A.B., Redley B., de Courten B., Manias E. (2021). Potentially inappropriate prescribing and its associations with health-related and system-related outcomes in hospitalised older adults: A systematic review and meta-analysis. Br. J. Clin. Pharmacol..

[10] General Authority for Statistics Population Estimates. https://www.stats.gov.sa/sites/default/files/POP%20SEM2021E.pdf.

[11] Bao Y., Shao H., Bishop T.F., Schackman B.R., Bruce M.L. (2012). Inappropriate medication in a national sample of US elderly patients receiving home health care. J. Gen. Intern. Med..

[12] Fialová D., Topinková E., Gambassi G., Finne-Soveri H., Jónsson P.V., Carpenter I., Schroll M., Onder G., Sørbye L.W., Wagner C. (2005). Potentially inappropriate medication use among elderly home care patients in Europe. JAMA.

[13] Fick D.M., Semla T.P., Steinman M., Beizer J., Brandt N., Dombrowski R., DuBeau C.E., Pezzullo L., Epplin J.J. (2019). American Geriatrics Society 2019 updated AGS Beers Criteria^®^ for potentially inappropriate medication use in older adults. J. Am. Geriatr. Soc..

[14] Joint Formulary Committee (2022). BNF 83 (British National Formulary) March 2022.

[15] Levy H.B., Marcus E.-L., Christen C. (2010). Adverse reactions/medication safety: Beyond the beers criteria: A comparative overview of explicit criteria. Ann. Pharmacother..

[16] Lisowska A., Czepielewska E., Rydz M., Dworakowska A., Makarewicz-Wujec M., Kozłowska-Wojciechowska M. (2022). Applicability of tools to identify potentially inappropriate prescribing in elderly during medication review: Comparison of STOPP/START version 2, Beers 2019, EU (7)-PIM list, PRISCUS list, and Amsterdam tool—A pilot study. PLoS ONE.

[17] Sharma R., Arora M., Garg R., Bansal P. (2020). A closer look at the 2019 Beers criteria. Drugs Ther. Perspect..

[18] Alshammari H., Al-Saeed E., Ahmed Z., Aslanpour Z. (2021). Reviewing potentially inappropriate medication in hospitalized patients over 65 using explicit criteria: A systematic literature review. Drug Health Patient Saf..

[19] Campanelli C.M. (2012). American Geriatrics Society updated Beers Criteria for potentially inappropriate medication use in older adults. J. Am. Geriatr. Soc..

[20] Fick D.M., Cooper J.W., Wade W.E., Waller J.L., Maclean J.R., Beers M.H. (2003). Updating the beers criteria for potentially inappropriate medication use in older adults—Results of a US consensus panel of experts. Arch. Intern. Med..

[21] Abdelwahed A.A., El-Dahiyat F., Aljawamis D., Al Ajimi J., Bin Rafeea K.J. (2021). Potentially inappropriate medications in older adults according to Beers criteria 2019: Prevalence and risk factors. Int. J. Clin. Pract..

[22] Hasan S.S., Burud I.A.S., Kow C.S., Rasheed M.K., Chan K.S.C., Tay P.K., Ahmed S.I. (2021). Use of potentially inappropriate medications among older outpatients and inpatients in a tertiary care hospital in Malaysia. Int. J. Clin. Pract..

[23] Naughton M., Moriarty F., Bailey J., Bowen L., Redmond P., Molokhia M. (2022). A systematic review of the prevalence, determinants, and impact of potentially inappropriate prescribing in middle-aged adults. Drugs Ther. Perspect..

[24] Kroon D., Steutel N.F., Vermeulen H., Tabbers M.M., Benninga M.A., Langendam M.W., van Dulmen S.A. (2021). Effectiveness of interventions aiming to reduce inappropriate drug prescribing: An overview of interventions. J. Pharm. Health Serv. Res..

[25] Ilić D., Bukumiric Z., Jankovic S. (2015). Impact of educational intervention on prescribing inappropriate medication to elderly nursing homes residents. Srp. Arh. Celok. Lek..

